# Curvularia Pneumonia Presenting as a Mass-Like Lesion

**DOI:** 10.7759/cureus.25933

**Published:** 2022-06-14

**Authors:** Eby Thekkedath, Zachary Burden, Scott Steinberg, James Cury

**Affiliations:** 1 Internal Medicine, University of Florida College of Medicine, Jacksonville, USA; 2 Pulmonary and Critical Care Medicine, University of Florida College of Medicine, Jacksonville, USA

**Keywords:** pulmonary infection, dematiaceous fungi, phaeohyphomycosis, fungal pneumonia, curvularia

## Abstract

*Curvularia* species of fungi are ubiquitous and mostly comprise plant or soil residents. Rarely pathogens, they are found in tropical and subtropical climates. On rare occasions, these fungi can be of clinical significance and lead to a variety of disease processes, mainly in immunocompromised individuals. Most infections are limited to allergic fungal rhinosinusitis; however, disseminated disease and invasive infections have been increasingly reported. There exist no therapeutic guidelines for invasive *Curvularia* infections currently, but amphotericin and various azoles have been used with varying degrees of success. We present a unique case of an immunocompetent 44-year-old female who presented with symptoms concerning for pneumonia and was found to have a mass-like lesion in the lung concerning for malignancy. Biopsy and histopathology of the lesion were consistent with invasive *Curvularia* pulmonary infection. We reviewed this case in the setting of reported literature concerning *Curvularia* with an emphasis on the epidemiology, pathology, diagnosis, and emerging management protocols of invasive *Curvularia* infections.

## Introduction

*Curvularia* fungal infections are rarely seen in humans. There are currently over 100 identifiable species within the *Curvularia* genus, but only a few have been reported to cause diseases. The primary species associated with human infections include *C. aeria, C. lunata, and C. geniculata*. *Curvularia* species are classified into a group of pigmented fungi called dematiaceous fungi and are characterized by their brown/black pigment and rapid growth in colonies [1]. Human colonization and infections are usually acquired from trauma or inhalation; however, they can also spread via contaminated products such as gardening tools that penetrate the skin. Generally speaking, dematiaceous fungi like *Curvularia* species have low inherent virulence; however, fungal pulmonary infections are increasing in a growing immunosuppressed population. Pulmonary infections with these fungal species are very rare in immunocompetent individuals and specific risk factors in this population have yet to be identified. Clinical manifestations can vary from asymptomatic pulmonary nodules to PNA and endobronchial lesions causing hemoptysis [2]. There are no conclusive guidelines currently available for the management due to the rarity and infrequent presentations of infection. However, current recommendations in the reported literature suggest azole therapy with amphotericin only to be used in rare circumstances of dissemination or CNS infection [3].

This article was previously presented as a case report poster at the 2020 CHEST Annual Meeting held from October 18 to 21, 2020.

## Case presentation

**Figure 1:**
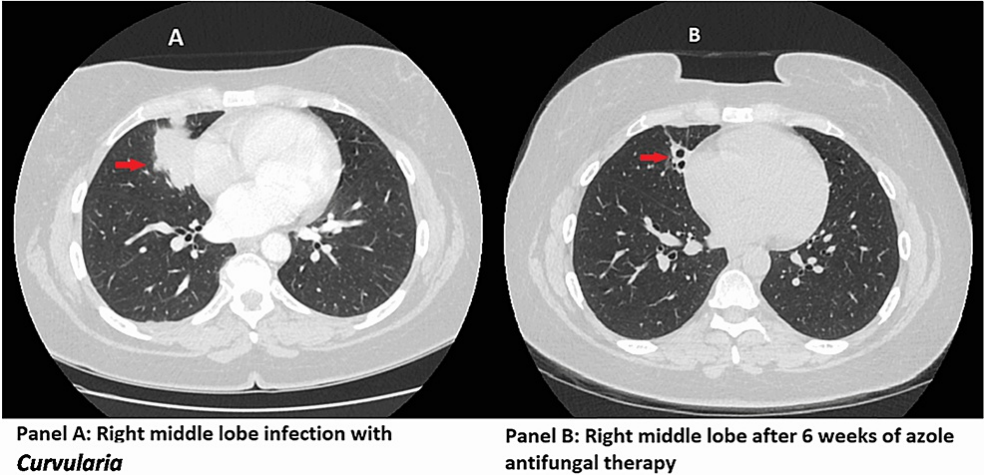
CT chest imaging CT: computed tomography

A 44-year-old female with a past medical history of 15 pack-years cigarette use but currently with eight years of abstinence presented to her primary care center with shortness of breath, cough, and purulent sputum for over three to four weeks. The patient was initially diagnosed with pneumonia and treated with levofloxacin, but her symptoms worsened. She presented to an outside hospital and a CT scan of her chest revealed a 5.8-cm right middle lobe mass-like lesion concerning for malignancy (Figure 1A). The patient then underwent bronchoscopy with transbronchial biopsy of the right middle lobe and bronchial wash from the right middle lobe. The pathology from the transbronchial biopsy of the right middle lobe was non-diagnostic and the cultures from the bronchial wash were initially unrevealing.

The patient then underwent a CT-guided percutaneous core biopsy of the right middle lobe. Pathologic examination of this specimen revealed granulomatous and eosinophilic pneumonitis with rare fungal forms identified. Subsequently, the patient’s bronchial wash culture demonstrated fungal growth with *Curvularia* species. She underwent treatment initially with fluconazole for two weeks. Once susceptibilities returned from the reference laboratory, the patient was switched to posaconazole for a total of six weeks of therapy. After the completion of therapy, the patient had resolution of her symptoms, and a repeat CT scan of her chest revealed near-complete resolution of the lesion with mild residual scarring and traction bronchiectasis (Figure 1B).

## Discussion

*Curvularia* species of fungi are ubiquitous and mostly constitute plant or soil pathogens found in tropical and subtropical climates. To date, more than 10 unique species have proven to be pathogens of clinical significance. Documented human mycoses due to *Curvularia* infection include onychomycosis, endocarditis, invasive sinusitis, keratitis, systemic infection, and cerebral phaeohyphomycosis. Generally speaking, phaeohyphomycosis is an infection caused by dematiaceous molds, of which *Curvularia* is one of more than 150 species pathogenic in humans [3]. However, most phaeohyphomycotic infections are superficial, cutaneous, or subcutaneous. Rarer, invasive infections involving an organ system and or disseminated infection can occur in immunocompromised hosts. Even bronchopulmonary infections secondary to *Curvularia* have been associated with either an immunocompromised host or those with significant underlying lung pathologies such as bronchiectasis or cystic fibrosis. Hence, this case was highly unusual given the patient's lack of prior medical history, immunocompetent state, and the absence of any underlying lung pathology. It is important to note that infections can be seen adjacent to neoplasms in suspected lung malignancy. Diagnosis is very challenging as the primary mode of diagnosis for all agents of phaeohyphomycosis is fungal culture and histopathologic analysis of tissue aided by Fontana-Masson stain. However, even with these modalities, histopathologic interpretation can be difficult as different pathologic features can be produced in tissues by the same fungus due to suspected host factors [4]. Management and treatment vary based on the species, site, and extent of infection, as well as susceptibilities to antifungal treatment. Amphotericin B is effective against most species; however, aggressive therapy is often not required unless a disseminated infection is suspected, in which case the mortality rate is >70% regardless of therapy [5]. Other antifungals with good coverage against most cases of phaeohyphomycosis include voriconazole, itraconazole, and posaconazole. The *Curvularia* species isolated in our case was susceptible to amphotericin, itraconazole, and posaconazole but resistant to voriconazole.

## Conclusions

*Curvularia* pneumonia in immunocompetent patients is very rare and the underlying risk factors predisposing to these infections are still unclear. We presented a rare case of phaeohyphomycosis pneumonia presenting as a unilateral mass with complete resolution after identifying the rare pathogen and providing susceptibility-guided treatment.
